# Immune Checkpoint Inhibitors in Cancer Therapy—How Can We Improve Clinical Benefits?

**DOI:** 10.3390/cancers15030881

**Published:** 2023-01-31

**Authors:** Constantin N. Baxevanis

**Affiliations:** Cancer Immunology and Immunotherapy Center, Cancer Research Center, Saint Savas Cancer Hospital, 11522 Athens, Greece; costas.baxevanis@gmail.com; Tel.: +30-2106409380

Immune checkpoint inhibitors (ICIs) are in the spotlight of cancer treatment by increasing the probability for long-term survival in patients with metastatic disease and by considerably prolonging progression-free survival in patients at early disease stages. Over the last decade, the FDA has approved a number of ICIs, opening new avenues for the therapeutic treatment of a variety of cancer types. However, despite remarkable clinical results, the majority of patients either undergo therapeutic failure or develop immunotherapy resistance. Therefore, it is important to identify biomarkers predicting clinical response and to increase the success rates of ICIs-based therapies by proposing combinatorial treatments. The major aim of this Special Issue of *Cancers* is to shed more light on the clinical significance of biomarkers by exploring the predictive characteristics of patients’ tumor microenvironment (TME) and discuss therapeutic modalities which intend to modify this to a milieu favoring responses to immune checkpoint inhibition (ICI).

The review by Hudry et al. [1] highlights the prognostic role of intratumoral CD8+ T cells (IT-CD8+) for clinical efficacy during treatment with ICIs in epithelial ovarian cancer (EOC) and their relationship to programmed cell death-ligand 1 (PD-L1) expression. Results from various studies evaluating high-grade serous ovarian carcinoma revealed a favorable role for improved overall survival (OS) when negative PD-L1 expression in tumor cells was combined with high IT-CD8+, whereas the reverse combination (i.e., positive PD-L1 expression in tumors and low IT-CD8+) revealed the shortest OS. The expression of PD-1 in IT-CD8+ was also linked to favorable prognosis. Importantly, the authors also discussed data from various studies proposing the combination of neoadjuvant chemotherapy (NACT) with ICI. To this end, they reviewed results from patients undergoing NACT which showed an increase in antitumoral immune response based on high densities of natural killer cells, increased cytotoxic, Th1 and IFNγ signatures, enhanced levels of PD-L1 expression in tumor cells and decreased frequencies of regulatory cells in the TME post-NACT. These results demonstrated an antitumor immune response to NACT, as well as reaction of tumor cells through the expression of PD-L1. Therefore, ICI could improve clinical outcomes after the first line of chemotherapy. In addition to PD-1/PD-L1, the detection of other immune checkpoints in the TME of patients with EOC, such as T-cell immunoglobulin, mucin domain-containing protein 3 (Tim-3), lymphocyte activating gene 3 (LAG-3), and cytotoxic T-lymphocyte associated protein 4 (CTLA4), were also suggestive for combinatorial ICI. In the review by Nishimura et al. [2], the authors examine the inverse situation, namely the clinical efficacy of second-line chemotherapy, in non-small cell lung cancer patients who had previously received ICI. Patients were grouped into those who had received docetaxel alone or combined with a monoclonal antibody targeting the vascular endothelial growth factor receptor–2 (ramucirumab), previously treated or non-treated with ICIs. There was an almost 2.5-fold increase in the overall response rate in the group of patients receiving docetaxel/ramucirumab pretreated with ICIs as compared to the ICIs-untreated group (57.1% vs. 20%, respectively). In addition, the docetaxel group showed a 3-fold increase in the overall response rate of patients pretreated with ICIs vs. untreated patients (15.4% vs. 5%, respectively). The elimination of cells negatively regulating antitumor reactivity (e.g., myeloid-derived suppressor cells and/or regulatory T-cells) increased neoantigen expression followed by antigen cross-presentation, disruption of tumor stroma with subsequent increased immune penetration, and upregulation of death receptors in tumor cells.

The review by Oronsky et al. [3] proposes the combination of ICIs with oncolytic viruses (OVs), genetically engineered to express genes activating both innate and adaptive immune response to target tumors. Besides the expression of transgene-encoded proteins with immunomodulatory properties, the OVs can directly eliminate cancer cells, thereby stimulating the release of danger signals and tumor-associated or tumor-specific antigens, thus acting as an in situ vaccine. In this way, OVs function to turn immunologically “cold” TMEs into “hot” ones supporting the generation of robust antitumor responses during ICI. The authors emphasize that the full potential of OVs in combination with ICIs has not yet been realized in large phase III trials, which suggests the need for improvements in the design of OVs. Such advances should be made to facilitate targeting of multiple immune-stimulating steps of the cancer-immunity cycle, which will modify the immune TME in a way to enable durable ICIs-induced responses in combination with other OVs and targeted therapies counteracting suppressor mechanisms and angiogenesis. 

The next review by Agnarelli et al. [4] discusses the role of immunotherapy in the clinical management of advanced gastric cancer (GC) based on phase III clinical trials in the first line of treatment, or beyond, whereby increasing PD-L1 expression has been linked to improved clinical outcomes with ICI (anti-PD-1/anti-PD-L1). Moreover, besides those expressing PD-L1, there are additional molecular subgroups of GC which have been reported to respond to immunotherapies, including microsatellite instability high (MSI-H)/mismatch repair deficient (MMR-D) ones with high tumor mutational burden or with chronic EBV infection. However, despite the presence of these favorable biomarkers, there is still a sizeable number of GC patients who do not adequately respond or develop resistance following response to immunotherapies. To this end, the authors discuss large phase III clinical studies aiming at the optimization of clinical results from immunotherapies through the combined use of ICIs with chemotherapies. The authors also discuss the immunomodulatory effects induced by *H. Pylori* infection and its impact on the efficacy of antitumor immune responses induced by ICIs. Given the up-regulation of PD-L1 in cases of chronic *H. Pylori* infections, it is conceivable that *H. Pylori* may affect clinical outcomes with anti-PD-1 or anti-PD-L1 in patients with GC. HER-2/neu is also being considered as a favorable candidate biomarker because, when overexpressed, it could be targeted with Trastuzumab inducing antibody-dependent tumor cell cytotoxicity. In addition, HER-2/neu overexpression in PD-L1+ GC cancers would support the combined treatment with anti-PD-1/anti-PD-L1 and Trastuzumab. Nevertheless, a possible favorable synergistic effect between ICIs and Trastuzumab or a possible correlation between *H. Pylori* and the effectiveness of PD-1/PD-L1 inhibitors has been studied in a limited number of small-sized clinical trials, so there is no solid evidence yet for the role of *H. Pylori* or HER-2/neu as predictive biomarkers of response to ICI in GC.

From the studies discussed above, it is plausible that the resistance mechanisms employed by tumor cells provide a formidable obstacle for the widespread success of ICI. Therefore, understanding the underlying mechanisms resulting in immunotherapy resistance will help to narrow the ICI-resistant patient population. In their review article, Lao et al. [5] discuss various therapeutic modalities which, alone or in combination, will help to reduce resistance to immunotherapies utilizing ICIs. In this direction, they propose two main strategies resulting in inflamed tumors with reinvigorated endogenous antitumor immunity. The first strategy aims at increasing the recruitment of immune lymphocytes in the tumor compartments and at the same time enhancing T-cell recognition for the destruction of tumor cells. The second strategy is based on inducing immunogenic tumor cell death intratumorally for improving immunogenicity and generating robust endogenous antitumor reactivity. To this end, they propose combinations of ICIs with (i) cytokines/chemokines for increased T-cell infiltration in the tumor; (ii) CAR T-cells for improving tumor cell recognition and destruction; and (iii) OVs, radiotherapy, photodynamic therapy, hyperthermia, and chemotherapeutic drugs for inducing immunogenic cell death and improving immunogenicity. The combination of ICIs with therapeutic vaccines provides a useful combinatorial treatment for increasing immune cell infiltration in the tumor, followed by enhanced immune cell-mediated tumor cell destruction. Increased genomic methylation may also restore responses to ICI by counteracting resistance mechanisms. Indeed, hypomethylation has been implicated in the generation of mechanisms resulting in tumor evasion from immune surveillance and resistance to ICI [6]. S-adenosylmethionine (SAM) is a ubiquitous methyl donor which targets DNA hypomethylation and blocks DNA demethylation, resulting in the downregulation of several essential oncogenes and pro-metastatic genes including matrix metalloproteinase-2 and 9 (MMP-2/9) and FOXP3 gene, reducing tumor growth and metastasis, and diminishing the immunosuppressive capacity of Tregs [7,8]. Although the luminal B subtype has the lowest response rates to ICI and the highest capacity to form bone metastasis compared to other subtypes of breast cancer, still, this molecular subtype may have certain immunological features that could increase sensitivity to ICI, including the expression of immune checkpoints, higher mutational load, and immune infiltration in the TME [9,10,11,12]. In their research study, Mehdi et al. [13] investigated the combination of SAM with ICIs to enhance responses to immunotherapy in an experimental mouse model using a transplantable luminal B tumor cell line. They were able to show that the combination treatment (as opposed to single treatment either with SAM or ICIs) produced the highest reduction rate in tumor growth and progression and reduced metastasis to the lungs and bones, proposing that this combined treatment could also be effective in patients suffering from this subtype of breast cancer. In their study, Giatromanolaki et al. [14] provide data to support the therapeutic targeting of the CD47/signal regulatory protein alpha (SIRPα)-axis during neoadjuvant treatment of operable non-small cell lung cancer. They determined that the majority of tumor cells in these patients overexpress the CD47 ligand which was directly associated with SIRPα expressed by tumor-associated macrophages and poor postoperative prognosis. Moreover, their analyses did not show any associations between CD47/SIRPα and PD-1/PD-L1 expression. Interestingly, there was a direct relation of CD47 expression with poor infiltration by immune lymphocytes and, most importantly, with increased frequencies of FOXP3+ regulatory T cells. The next article, by Noji et al. [15], delves into the significance of comprehensive genomic profiling (CGP) for providing accurate information on cancer-related genetic aberrations and establishing a notable relationship between treatment response and oncogene profiling data in ICI-treated patients with Head and Neck Squamous Cell Carcinoma (HNSCC). The authors confirmed data from other studies, namely that high tumor mutational burden (TMB-high) predicts long-term responses to ICI. However, TMB-high was observed only in 3% of SCC cases, which confers no practical value to this biomarker. Additionally, in some patients with TMB-low or -intermediate, a shrinkage in tumor load was observed initially, followed by tumor progression, suggesting that resistance may have been acquired during ICI. This implied that HNSCC patients with non-high TMB may benefit from ICI combined with drugs that counteract acquired immune resistance. To this end, by applying CGP, the authors demonstrate that *CCND1* amplification (*CCND1* encodes cyclin D1 which regulates the retinoblastoma protein activity and cell-cycle progression) was strongly associated with diminished reactivity to ICI and a poor prognosis independent of TMB levels and PD-L1 expression. ICI combination therapies in Head and Neck Cancers have been comprehensively reviewed by Ettl et al. [16]. The authors provide an overview of the combination of ICIs with various conventional and novel therapeutics aiming at (i) targeting inhibitory cellular mechanisms to reinforce antitumor immunity in the TME; (ii) potentiating endogenous T-cell responses against the tumor by blocking immunosuppressive factors such as IL-10, TGFβ, VEGF, COX2, and tumor glucose or glutamine metabolism; (iii) promoting T-cell expansion, function, and survival; and (iv) facilitating tumor regression by triggering a cytotoxic T cell-response, reducing the number of myeloid-derived suppressor cells, tumor-associated macrophages, and Tregs and activating dendritic cells. The authors also review the combination of ICIs with other immunotherapeutic modalities such as cellular therapies and active immunization as well as with immunomodulators blocking oncogenic pathways. Given the central role of CD8+ T cells in mechanistic pathways initiated by ICIs, it is of great importance to apply reliable methods for their quantitation. Ramlee et al. [17] propose “Radiomics” as a non-invasive method for estimating CD8+ tumor infiltrating lymphocytes (TILs) alternatively to the measurement of CD8+ TILs density relying on tissue sampling. The authors remark that the identification of imaging features, representing a “radiomic signature”, linked to CD8+ TILs could form a novel non-invasive method for assessing treatment outcomes, complementary to routine patient management. 

Besides promoting clinical efficacy, ICI cause many immune-related adverse events. In their review article, Shalata et al. [18] discuss ICI-induced cutaneous adverse events with a focus on Bullous Pemphigoid (BP) and proposed treatments mostly including topical or systemic steroids. Furthermore, they discuss differences in kinetics for BP onset following treatments with different ICIs and also propose putative mechanisms to explain why some patients develop BP and others do not. Fatigue is one of the most frequent adverse effects caused by ICIs. Physical activity has been shown to help ICIs-treated cancer patients to overcome adverse effects also including fatigue. In their scoping review, Shaver et al. [19] included clinical trials of patients being treated with ICIs for clarifying the concept of physical activity as concurrent therapy to ICIs, and to describe the outcomes of physical activity as concurrent therapy to ICIs. The authors also discuss pre-clinical studies which indicate that the combination of physical activity and ICI could provide significant benefits for patients by decreasing fatigue and potentiating the endogenous antitumor immunity thus providing the means for improved clinical benefits. Antibiotics have a negative impact on cancer immunotherapy; however, their effect on the overall immune function is unclear. In their study, von Itzstein et al. [20] aim to identify differences in immune parameters following antibiotic exposure by evaluating therapeutic responses, and serum cytokines and antibodies at baseline and six weeks after ICI initiation. They determine that prior antibiotic administration caused significant changes in the levels of certain circulating antibodies. They also observe differences in serum IFN-γ, IL-8, and macrophage inflammatory proteins. Although such differences were not associated with clear biological or clinical significance, they propose the design of future studies for more precisely characterizing the mechanistic pathways induced by antibiotics on immune physiology and clinical outcomes. 

This Special Issue of *Cancers* provides new information about mechanisms which reduce the clinical efficacy of ICIs in the context of multiple different cancer types. Novel treatment options combining ICIs with other ICIs, immunomodulators, vaccines, cellular and targeted therapies, chemotherapies and radiotherapies are also included. I hope that this knowledge will stimulate further translational and clinical research and therapeutic trials with ICIs to maximize their clinical benefits in various human cancers.
